# The use of rapid review methods in health technology assessments: 3 case studies

**DOI:** 10.1186/s12874-016-0216-1

**Published:** 2016-08-26

**Authors:** Eva Kaltenthaler, Katy Cooper, Abdullah Pandor, Marrissa Martyn-St. James, Robin Chatters, Ruth Wong

**Affiliations:** School of Health and Related Research (ScHARR), University of Sheffield, Regent Court, 30 Regent Street, Sheffield, S1 4DA UK

**Keywords:** Rapid review methods, Health technology assessment, Systematic review

## Abstract

**Background:**

Rapid reviews are of increasing importance within health technology assessment due to time and resource constraints. There are many rapid review methods available although there is little guidance as to the most suitable methods. We present three case studies employing differing methods to suit the evidence base for each review and outline some issues to consider when selecting an appropriate method.

**Methods:**

Three recently completed systematic review short reports produced for the UK National Institute for Health Research were examined. Different approaches to rapid review methods were used in the three reports which were undertaken to inform the commissioning of services within the NHS and to inform future trial design. We describe the methods used, the reasoning behind the choice of methods and explore the strengths and weaknesses of each method.

**Results:**

Rapid review methods were chosen to meet the needs of the review and each review had distinctly different challenges such as heterogeneity in terms of populations, interventions, comparators and outcome measures (PICO) and/or large numbers of relevant trials. All reviews included at least 10 randomised controlled trials (RCTs), each with numerous included outcomes. For the first case study (sexual health interventions), very diverse studies in terms of PICO were included. P-values and summary information only were presented due to substantial heterogeneity between studies and outcomes measured. For the second case study (premature ejaculation treatments), there were over 100 RCTs but also several existing systematic reviews. Data for meta-analyses were extracted directly from existing systematic reviews with new RCT data added where available. For the final case study (cannabis cessation therapies), studies included a wide range of interventions and considerable variation in study populations and outcomes. A brief summary of the key findings for each study was presented and narrative synthesis used to summarise results for each pair of interventions compared.

**Conclusions:**

Rapid review methods need to be chosen to meet both the nature of the evidence base of a review and the challenges presented by the included studies. Appropriate methods should be chosen after an assessment of the evidence base.

## Background

Systematic reviews have long been a key component of evidence based medicine. The use of methods to expedite systematic reviews is ever increasing due to time and resource constraints as well as policy maker and clinical demand. Systematic reviews typically take at least 12 months to conduct with rapid reviews taking between 3 weeks and 6 months [1]. Although the use of rapid review methods is increasing, there is little agreement as to how they are defined and what methodologies should be used [2]. Tsertsvadze et al. [3] describe three ways in which systematic reviews may be done more quickly: 1) process parallelisation using several reviewers to perform tasks in parallel; 2) innovative technologies to assist with tasks such as study selection, data extraction and risk of bias assessment and 3) modification of systematic review methodologies such as restricting or bypassing one or more steps in the process. Modifications to standard systematic review methods may include: highly refined research questions, limited searching [4], reduced number of reviewers for sifting and data extraction, restricted study design and limited quality assessment [1] and updating existing reviews [5]. The level and detail of analyses and synthesis is often reduced and existing systematic reviews may be summarised [6] or evidence summaries produced [7, 8]. Rapid review methods may be described as those that seek to reduce the time associated with systematic review methods in a way that will have the least impact on the validity or utility of the results. The reviewer must decide which modifications to standard methods will do this, given review objectives, time constraints and other challenges. Rapid reviews are particularly important in the field of health technology assessment (HTA) where they are used to support informed decision making [7]. Rapid review methods are not unique to HTA, although the need for timely evidence to underpin the assessment of new technologies makes them particularly relevant in this context.

There are many inherent limitations associated with the use of rapid reviews such as the risk of introducing publication bias due to reduced searching. Grant and Booth [9] suggest that by limiting quality assessment and appraisal of evidence, disproportionate emphasis may be placed on poorer quality studies and lack of attention to synthesis may overlook inconsistencies or contradictions in the data. In addition, rapid reviews are less likely to use external experts and peer review and therefore they have potentially less scrutiny from clinical and methodological experts [4]. Methodological details of the rapid review process are often not mentioned or poorly described in reviews that use these methods [10]. In addition, the limitations associated with the chosen rapid review approach are frequently not discussed [2].

Recommendations for conducting rapid reviews include the need for replicability and transparency of methods [5]. This includes the need for a clearly stated research question, inclusion criteria, search strategies, inter-rater agreement process, data extraction and synthesis methods and conclusions. Also essential is a description of the limitations of the chosen methods. A recent summit of evidence synthesis experts developed a rapid review research agenda which included the need for a rapid review taxonomy and definitions and the need for methodological guidelines [11]. There is little guidance currently available regarding the most suitable methods to use and no indication as to whether all methods are suitable for all rapid reviews. Reviewers are faced with a dilemma as to which rapid review methods to use to best suit the evidence base available and the time constraints of the review they are undertaking.

The National Institute for Health Research (NIHR) within the United Kingdom commissions rapid reviews in the form of short reports on a range of topics. We present three case studies of recent rapid reviews undertaken for the NIHR programme undertaken by the authors of this paper. These reviews were each undertaken within 8–12 weeks (from agreement of the final protocol to delivery of draft report) and all three reviews included almost entirely randomised controlled trials (RCTs) due to the limited time available to conduct the reviews. Three reviews from the same programme were chosen as they used identical processes for developing research questions, adherence to PRISMA reporting standards, report templates and peer review. Differences between the three reviews regarding the rapid review methods used arose from the differing evidence bases and challenges for each review. In this paper we present three distinctly different approaches to rapid reviewing and propose issues to consider when selecting appropriate rapid review methods for use in health technology assessments.

## Methods

The three most recently completed NIHR rapid reviews undertaken at the School of Health and Related Research, University of Sheffield, United Kingdom were included in these analyses. The three reports included in the analyses were:

- The effectiveness of sexual health interventions for people with severe mental illness (SMI): a systematic review [12].
- Interventions to treat premature ejaculation: a systematic review short report [13].
- Psychological and psychosocial interventions for cannabis cessation in adults: A systematic review short report [14].

These three reviews were chosen as they had distinctly different evidence bases requiring different approaches to rapid review, thus enabling us to explore a range of rapid review approaches. The commissioning brief or questions to be answered by the reviews were set by the NIHR and it was a requirement that all interventions listed in the commissioning brief be included in the reviews. The protocols for all three reviews were developed from the NIHR commissioning briefs, peer reviewed, agreed with NIHR and registered on the PROSPERO website and published on the NIHR website. All three reviews were undertaken by at least two experienced systematic reviewers with an experienced information specialist undertaking the literature searches. All three reviews had extensive searching, data extraction and quality assessment, intensive input from clinical experts and all underwent peer review of the draft report by independent external reviewers as part of the standard NIHR peer review processes.

The reviews all adhered to PRISMA reporting standards and are all published as part of the NIHR monograph series. For the present analyses, data were extracted from each of the short reports on the following variables:

- Rapid review methods used (reporting of outcomes, synthesis methods used).
- Research question and aims.
- Number of interventions and comparators.
- Number of included studies.
- Perceived reviewing challenges.
- Rationale for choice of review method in each case.
- Reported strengths of rapid review methods.
- Reported weaknesses of rapid review methods.

These variables were determined by the whole research team as they were deemed important in order to understand the approach and challenges of the chosen methods in each review. The authors of each of the three reviews contributed to the data extraction and interpretation for the reviews they authored.

## Results

The three reviews included in these analyses all used rapid review methods due to the short time frame of this HTA process. The rapid review methods used in all three reviews were: the use of a focussed research question, partial double sifting of titles and abstracts and partial double data extraction by a second reviewer. Two reviews included only RCTs or existing systematic reviews of RCTs [12] [14] while one review also included one non-randomised trial [13]. The quality of the included reviews has been assessed using the AMSTAR checklist [15] as shown in Table 1. Although rapid review methods were used in these reviews they are of relatively high quality according to the AMSTAR checklist.

**Table 1:** AMSTAR assessment of the three reviews

Question	Review 1 Sexual health	Review 2 Premature ejaculation	Review 3 Cannabis cessation
1. Was an 'a priori' design provided?	Yes, published protocol with research questions and inclusion criteria.	Yes, published protocol with research questions and inclusion criteria.	Yes, published protocol with research questions and inclusion criteria.
2. Was there duplicate study selection and data extraction?	Yes All abstracts and full text articles assessed by two reviewers, data extracted by one reviewer, checked by another.	Yes (partial) Titles and abstracts of citations identified by the searches were screened for potentially relevant studies by one reviewer and a subset checked by a second reviewer (and a check for consistency undertaken). Full texts were screened by two reviewers. One reviewer performed data extraction of each included study. All numerical data were then checked against the original article by a second reviewer. Any disagreements were resolved by a third reviewer.	Yes (partial). Titles and abstracts of citations identified by the searches were screened for potentially relevant studies by one reviewer and a 10 % sample checked by a second reviewer (and a check for consistency undertaken). Full texts were screened by two reviewers. One reviewer performed data extraction for each included study. All numerical data were checked against the original article by a second reviewer and any disagreements were resolved through discussion.
3. Was a comprehensive literature search performed?	Yes comprehensive searching reported.	Yes comprehensive searching reported.	Yes comprehensive searching reported.
4. Was the status of publication (i.e. grey literature) used as an inclusion criterion	Yes, Grey literature was searched, non-English papers excluded	Yes, some Grey literature was searched, non-English papers excluded	Yes, Grey literature was searched, non-English papers excluded
5. Was a list of studies (included and excluded) provided?	Yes tables of included and excluded studies both included	Yes tables of included and excluded studies both included	Yes tables of included and excluded studies both included
6. Were the characteristics of the included studies provided?	Yes	Yes	Yes
7. Was the scientific quality of the included studies assessed and documented?	Yes	Yes	Yes
8. Was the scientific quality of the included studies used appropriately in formulating conclusions?	Yes	Yes	Yes
9. Were the methods used to combine the findings of studies appropriate?	Yes	Yes	Yes
10. Was the likelihood of publication bias assessed?	No	No	No
11. Was the conflict of interest included?	Yes source of funding for included studies reported.	No sources of funding for included studies not reported	No sources of funding for included studies not reported
AMSTAR score	10/11	9/11	9/11

### Scoping searches and protocol development

Scoping searches, based on a simple search strategy, were done while developing the protocols to provide a quick overview of the potential evidence base in terms of existing reviews, approximate numbers of relevant studies, relevant interventions and comparators as well as types of outcomes reported. Scoping searches were essential in estimating the number of records that would be retrieved. This informed the development of the protocol and selection of rapid review methods. A brief summary of the comprehensive search strategies and search results for the three reviews can be found in Table 2. Study design filters were necessary in all three reviews so that the total number of records was manageable in the time frame.

**Table 2:** Search comparison table

	Sexual health of people with severe mental illness [12]	Premature ejaculation [13]	Cannabis cessation [14]
Scoping search date	November 2013	July 2013	January 2014
Design of included studies	RCTs comparing sexual health interventions with usual care for adults with severe mental illness (SMI)	RCTs of interventions for premature ejaculation (data extracted from existing reviews where available), or non-RCT evidence for any treatments where no RCTs exist	RCTs of psychological or psychosocial interventions for cannabis cessation in regular users of cannabis
Approaches to searching	Electronic database; contact experts; reference tracking	Electronic database; contact experts; reference tracking	Electronic database; contact experts; reference tracking
Sources to search	Four electronic databases (3 health and 1 subject specific); conference proceedings database; clinical trials registers; UK/International mental health organisations	Five electronic databases (2 health, 1 nursing and 1 multidisciplinary); conference proceedings databases; FDA or EMA websites	Four electronic databases (3 health and 1 subject specific); conference proceedings database; clinical trials registers; UK/International societies and organisations
Search strategy	Single strategy (17 statements for Medline) with RCT filter. Included all terms for SMI and focused mental health terms (schizophrenia, schizoaffective or bipolar) combined with various broad sexual health-related terms. Results from the Medline scoping search was compared to see if RCTs from a known Cochrane review had been missed	Focused strategy (8 statements for Medline) comprising terms for premature ejaculation with filters to identify RCTs, reviews or guidelines	Single strategy (28 statements for Medline) with filters to identify reviews and RCTs. Cannabis terms (comprehensive) combined with broad psychotherapy and behavioural therapy terms (derived from the scoping search). The strategy was developed from three known Cochrane reviews. Title and abstract keywords were incorporated into the strategy and checked to see if known RCTs included in the reviews have been captured by the strategy
Challenges	Not all mental health condition terms included. Individual sexually transmitted infections or behavioural terms not searched, due to the anticipated large number of irrelevant papers that would be identified	No specific intervention terms included due to large number of potential interventions. This increased potential for retrieval of a large number of records, but scoping searches indicated it would be a manageable number	Not all individually named psychotherapy or behavioural therapy terms searched due to the anticipated large number of irrelevant papers that would be identified
Records retrieved in Medline scoping search	RCTs filter 684	RCTs filter 521; systematic reviews filter 653; cohort studies filter 596; guidelines filter 9	RCTs filter 361; systematic reviews filter 36
Main search date	December 2013	August 2013	February 2014
Total records from main database search	2586	2283	1079

There was a process of iteration for each of the reviews in the form of specific questions from the reviewers to the policy makers to ensure that the proposed methods would meet the needs of the policy makers. In addition, the policy makers were able to comment on the draft protocols and revisions to the protocol incorporated in the final version. The approaches chosen were in part in order to meet the requirements of the policy makers as set out in the commissioning briefs to look at all relevant interventions.

Table 3 summarises the key characteristics of the three included short reports and the rapid review approaches adopted by the review teams.

**Table 3:** Summary characteristics of the three reviews, review challenges and approaches and strengths and limitations of chosen methods

Report and no. RCTs	Populations, interventions and comparators	Review challenges and approaches	Strengths and limitations of chosen method
Kaltenthaler et al. 2014 [12] Sexual health of people with severe mental illness 13 RCTs	Review aims: summarise effectiveness evidence, determine applicability in UK NHS setting and identify key areas for primary research. Population: people with severe mental illness Interventions: strategies to increase knowledge, assess and reduce sexual health risk, change behaviour and develop condom skills Comparators: educational sessions on HIV, money management or substance abuse, health promotion, wait list or no treatment	Challenges due to evidence base: • Wide variation in populations and settings (patients in psychiatric clinics, residential centres and homeless shelters) • Wide range of outcomes including: biological (sexually transmitted infections, pregnancy), behavioural (number of partners, uptake of services, use of contraception/condoms) and proxy (knowledge, attitudes, behaviours, facilitators and barriers etc.) Approaches: • Focussed definition of severe mental illness • Brief summary of results presented, narrative synthesis, grouping of results from included studies by outcome (biological, behavioural and proxy)	Strengths: • Enabled rapid synthesis of a disparate evidence base to ensure policy makers were aware of areas where evidence was available. This informed the design of relevant RCTs Limitations: • Quantitative data synthesis not generated for use by policy makers (only effect size by intervention and outcome) • In-depth narrative synthesis not possible • Non-RCT evidence excluded
Cooper et al. (2015) [13] Premature ejaculation 101 RCTs and 1 CT (65 RCTs from existing reviews and 36 new RCTs and 1 new CT reports)	Review aims: synthesise effectiveness evidence for behavioural, topical and systemic treatments. Population: men with premature ejaculation Interventions: topical anaesthetics, antidepressants, phosphodiesterase-5 inhibitors, opioid analgesics, behavioural therapies, acupuncture, Chinese medicine Comparators: placebo, wait list, other therapies	Challenges due to evidence base: • Very large number of RCTs (over 100) and existing systematic reviews covering wide range of interventions (several drug classes plus behavioural approaches) • Several existing systematic reviews Approaches: • Meta-analysis of primary outcome using data extracted from existing systematic reviews, with new primary study data added • Narrative synthesis of secondary outcomes	Strengths: • Meta-analysis able to be used for primary outcome (consistent primary outcome) • Use of data from existing reviews enabled meta-analysis of large dataset in shorter time Limitations: • Potential for data errors or synthesis errors in original reviews to be repeated in new report • Methodological quality of studies extracted from existing reviews not assessed separately • Although use of data from existing reviews saved some time, triangulation of data from multiple reviews was still time-consuming • Original RCT publications not revisited for data extraction and quality assessment.
Cooper et al. (2015) [14] Cannabis cessation 33 RCTs	Review aims: summarise effectiveness evidence for psychological and psychosocial interventions and identify key areas for primary research. Population: adults who use cannabis regularly Interventions: cognitive behavioural therapy, motivational interviewing, motivational enhancement therapy, supportive-expressive dynamic psychotherapy, social support groups, case management, contingency management (vouchers as incentive/reward) Comparators: waitlist, treatment as usual, other interventions, assessment only, education controls, written cannabis information, cannabis education	Challenges due to evidence base: • Wide variation in study populations (extent of cannabis dependence), interventions (type, duration) and comparators • Very little consistency in outcome measures, time points, and statistics reported • Large number of RCTs for a short report Approaches: • For each pair of interventions compared, narrative summary of outcomes reported and how many showed a statistically significant effect	Strengths: • Inclusive approach, covering a wide range of populations, interventions and outcomes • Included all psychosocial or psychological interventions undertaken in the adult, community dwelling population of cannabis users Limitations: • Detailed numerical outcome data not presented, since outcome measures and statistics reported were so disparate • Outcome measures in RCTs not converted to consistent measures to compare across studies as not feasible in timeframe

*CT* controlled trial

### Review 1 The effectiveness of sexual health interventions for people with severe mental illness (SMI) [12]

The aim of this review was to evaluate the effectiveness of sexual health interventions for people with SMI, determine their applicability to the UK NHS setting, and to identify key areas for primary research. Thirteen RCTs were included in the review. The challenges for this review were the inclusion of very diverse studies in terms of populations, interventions, comparators and reported outcomes. There was a large volume of non RCT, uncontrolled study evidence identified in the initial scoping searches which was not included as this was considered to be lower quality evidence. A narrative synthesis approach was chosen as meta-analysis was deemed impossible due to the considerable study heterogeneity. Difficulties were encountered with defining “severe mental illness” which includes different conditions (such as major depression) in some countries but not others.

The approach taken was to briefly report all relevant outcomes; grouped into categories including biological, behavioural and proxy (such as barriers and facilitators) categories. Information on effectiveness of interventions was presented by reporting the p-values for outcomes as reported in the individual studies as well as the study authors’ conclusions.

The strengths of this approach were that only higher quality evidence was presented; a thorough assessment of quality was undertaken and key details for each included study were readily accessible, particularly information on outcomes. By using a focussed and previously agreed definition of severe mental illness, only studies with populations directly relevant to the needs of the policy makers were included. Limitations of the chosen approach were the exclusion of non-RCT evidence meaning that some information might have been lost, particularly that related to the description of interventions. It was also only possible to report limited quantitative outcome data. However, due to the nature of the evidence base more extensive quantitative synthesis would most likely not have been possible. Our chosen approaches allowed us to fulfil the review objectives in that an overview of the effectiveness of the included interventions was provided, despite not being able to report full quantitative data. In addition, areas for future research and trial design were identified and information on applicability to the UK NHS was provided.

### Review 2 Interventions to treat premature ejaculation [13]

The aim of the review was to systematically review the evidence for the clinical effectiveness of behavioural, topical and systemic treatments for premature ejaculation. The main challenge for this review was the large number of interventions and very large number of relevant RCTs (over 100).

The approach taken was to use meta-analysis where appropriate using data extracted from existing systematic reviews. Data from newer primary studies were added to the meta-analyses. The review included 102 controlled trials. Data from 65 of these RCTs were extracted from existing reviews and 37 directly from additional RCT publications. Meta-analysis was possible as most studies used the same primary outcome (intra-vaginal ejaculatory latency time), in contrast to the other two short reports discussed here where outcome measures varied greatly across studies. Secondary outcomes were more varied and narrative synthesis was used for these. This approach had limitations as there was the potential for incorporation of synthesis errors from the original reviews. The methodological quality of studies extracted from the existing reviews was not assessed separately due to time constraints. Although the use of data from existing reviews saved some time, triangulation of data from multiple reviews was still time-consuming. The approaches chosen allowed us to provide up to date quantitative evidence on the effectiveness of a range of treatments for premature ejaculation.

### Review 3 Psychological and psychosocial interventions for cannabis cessation in adults [14]

The aim of the review was to systematically review the effectiveness of psychological and psychosocial interventions for cannabis cessation in adults who use cannabis regularly and to identify key areas for primary research. This review included 33 RCTs. The cannabis cessation review included input from a service user who had previously received similar interventions to those included in the review. This individual provided feedback on the review protocol and final report; specifically, the included interventions, outcome measures and the lay person summary. The challenges associated with this review included a wide variation in study populations, such as the extent of cannabis dependence, interventions (type, duration, group or individual) and comparators. There was also very little consistency with regard to outcome measures, time points of measurement and statistics reported. Broad inclusion criteria were used as the commissioners requested the review to be inclusive of all relevant evidence.

The approach taken was to present a narrative summary of outcomes reported for each intervention and comparison, stating how many showed a statistically significant effect. This enabled inclusion of the many relevant studies within the time constraints of the review. Due to the significant study heterogeneity, meta-analysis was not considered suitable. The approaches chosen in this review provided an overview of effectiveness of the interventions although it was not possible to report the quantitative data in full. This approach differed from that used in the first short report (sexual health interventions) in that due to the larger number of trials more limited outcome data was presented.

### Points to consider when determining a rapid review approach

Based on the diverse range of approaches used in these three rapid reviews we have identified issues that are important to reflect on when planning a rapid review. Table 4 outlines a checklist of some items that should be considered when choosing a rapid review method, based on these case studies.

**Table 4:** Checklist of items to consider when determining a rapid review approach

1. Assess the current evidence base-It is important to have an understanding of the evidence available before deciding which rapid review methods are most appropriate. Some points to consider are:
• Scoping searches - These are useful to estimate an approximate number of anticipated relevant studies.
• Existing systematic reviews - What are the search dates for the review (s) and the question answered by the review (s)? What is the methodological quality of the review(s)? This can be assessed using appropriate checklists. Did the review report a quality assessment of included studies? Consider using reported data to incorporate in a meta-analysis with newly identified studies.
• Summary of existing reviews - The findings of identified reviews could be presented plus a summary of any new studies using narrative synthesis.
2. Consider presentation of evidence-The complexity of the evidence base should be taken into account and an assessment made as to how much data should be presented and in what format. Some points to consider are:
• Meta-analysis Does the data support the use of meta-analysis? • Outcome data Can limited data on outcomes be reported? • Grouping of outcomes Can relevant outcomes be grouped to assist the reader in understanding the evidence base?
3. Ensure clear communication with policy makers - It is important that there is a common understanding between reviewers and policy makers as to the purpose of the review and the questions to be answered. Some points to consider are:
• In depth analysis Is it preferable to the policy maker to present an in depth analysis of a smaller selection of studies?
• Brief overview Is it preferable to the policy maker to present less information from a wider range of studies? • Highlight gaps in the evidence Will it be helpful to the policy maker to highlight gaps in the evidence to inform future research?
4. Clearly report rapid review methods used - It is crucial that the reader understands what rapid review methods have been used and the impact this may have on the findings of the review. Points to consider are:
• Description of methods-Have the rapid review methods been transparently reported highlighting differences from standard systematic review methods?
• Discussion of limitations Have the potential limitations and biases of chosen methods been described.

It is difficult to plan the review approach until there is a clear understanding of the type, amount and variation of evidence available and scoping searches are very useful for this. The incorporation of good quality existing systematic reviews may be considered. Presenting summaries of existing reviews and new studies or extracting data from existing reviews and incorporating into a meta-analysis with data from new studies are all options for consideration. Attention should be given to the most appropriate way to present the evidence, assessing both the amount of data to be presented and the most appropriate format. The level and type of evidence presented must be acceptable to both reviewers and policy makers. Clear communication with policy makers is crucial to ensure that the review being undertaken will address the question under consideration. This is especially important when using rapid reviews as the limited timeframe and available resources mean that there will be a trade-off between different aspects of the review such as thoroughness of searching, breadth of the research question and depth of analysis. It is essential that the methods used are clearly reported so that the reader is aware of the potential biases and limitations associated with chosen methods. Methods should be reported in enough detail so as to be reproducible and transparent.

## Discussion

This paper presents three distinctly different approaches to the rapid review of evidence to address a pre-defined research question within a limited time frame. The approaches included a brief summary and grouping of results for the sexual health review [12], meta-analysis incorporating data from existing reviews and new RCTs for the premature ejaculation review [13] and reporting of a narrative summary of significant outcomes for each intervention/comparator pair in the cannabis cessation review [14]. All three reviews included other rapid review methods such as the use of a focussed research question, partial double sifting of titles and abstracts and partial double data extraction. Only numerical data was double data extracted in the cannabis and premature ejaculation reviews. Narrative synthesis was used, as opposed to full qualitative synthesis of evidence, in two of the reviews (sexual health interventions [12] and cannabis cessation [14]). Cameron et al. [16] suggest that rapid reviews may benefit from the rigour of external peer review. All three rapid reviews included in this study had expert advisory panels and were peer reviewed by a minimum of two independent external reviewers. They were also deemed to be of relatively high quality using the AMSTAR checklist with scores of 10/11, 9/11 and 9/11.

Methods chosen for the rapid review of evidence must be both feasible and appropriate taking into account the requirements of the commissioners, the quantity and nature of the evidence and the time and resources available to do the review. The methods chosen for these three reviews were deemed acceptable to the commissioners as they approved the protocols where the methods were described in detail. The reasons for the commissioning of the reviews varied and this fed into the choice of review methods. For the sexual health review, the majority of evidence was from North America and it was not known how transferable this might be to the UK. The aim of the review was to inform the design of a UK based trial. The aim of the premature ejaculation review was to identify the most effective treatment option or combinations of treatment and the aim of the cannabis cessation review was to evaluate interventions for cannabis use and to identify important evidence gaps that might require further research. In all three reviews alternative methods could have been chosen such as the inclusion of fewer interventions, comparators or fewer outcomes, restriction of study design or the updating of existing systematic reviews. However, all of these options would have meant that either the needs of the commissioners were not met or that the high standards of the NIHR were not adhered to.

Policy makers require evidence to make decisions in a timely manner therefore choices need to be made as to how to select, analyse and present the evidence required. We chose the approaches presented here in part because other approaches were not acceptable to the policy makers (limiting the number of interventions considered). Research by Cameron et al. [16] comparing the findings from rapid vs full reviews found no difference in the essential conclusions reached by the reviews. Traditional systematic reviews are more likely to provide greater depth of information than rapid reviews [1] although rapid reviews have been found to meet the needs of knowledge users [8]. It is however important, as suggested by Schünemann and Moja [17], to ensure that guidelines for review conduct and reporting are adhered to. Boundaries between rapid and full systematic reviews are often blurred and many published systematic reviews use rapid methods.

The key factors identified in this study for consideration when selecting rapid review approaches include: an assessment of the evidence base, consideration of how to present the evidence, understanding the needs of the policy makers and adequate reporting of methods and their strengths and limitations. The range of reviewing options can then be considered by the reviewer once the size of the evidence base is ascertained including limiting the scope of the review [5] and streamlining processes for full text review and data extraction [1]. The incorporation of good quality existing systematic reviews is potentially very useful [6] [8] and can save valuable time and resources.

Previous research has also highlighted the importance of reporting and communication with policy makers. Without a full description of methods the direction and magnitude of any risk of bias cannot be fully assessed [1]. This has real implications for policy makers when making decisions based on rapid reviews and it is essential that limitations are clearly described. Varker et al. [18] advocate the use of a reporting template in rapid reviews in order to ensure a consistent approach. Reviews must be “fit for purpose” so that they reflect the knowledge needs of the commissioning body [10]. Hartling et al. [19] also stress the range of methods employed are both driven and supported by close and ongoing communication between the producer of the review and the end user, which is a very different context from most standard systematic reviews. Feedback from policy makers is crucial to ensure that the use of rapid review methods did not hinder decision making and to determine what approaches are useful to them and which are not.

There are several limitations to this research study. Only three rapid reviews were assessed, all from the same institution involving an overlap of reviewers. All three reviews were undertaken for the same HTA programme in the UK. Other programmes may have had other requirements and meant that other approaches were needed. Other reviewers from other organisations may have chosen different review methods. This limits the findings of the research. The authors of this paper were also the reviewers for the three case studies and therefore had in depth knowledge of the reasoning behind choices but other data extractors may have come to different conclusions compromising the replicability of this study. We deliberately looked at our own reviews so that we could provide an in depth description as to how and why decisions were made about which rapid review method to use, and what we found to be the strengths and limitations of these methods.

More research is needed in this area to provide guidance to reviewers to enable them to choose the most appropriate rapid review methods. One possible approach would be to select a sample of rapid reviews produced by a range of groups that used the general approaches outlined in these case studies and then use this sample to compare both within and between the three case approaches to help establish best practice in this area. Another possible future research approach would be to compare the rapid review methods used with a full systematic review in the same area. We have recently published a paper comparing our chosen rapid review methods with a full review for one of the interventions from the premature ejaculation review [20]. We found that in the topic area primary outcome data were the same whether the de novo rapid review method or a full review method were employed. However, due to limited reporting across reviews, quality assessment of all RCTs could only be undertaken as part of the full systematic review. Finally, future research could explore the comparison of different rapid review approaches on the same topic, bearing in mind that not all approaches will be relevant and feasible for every topic.

We did not receive feedback on whether or not our reviews met the needs of the commissioners, although this was requested. We have suggested this become part of the NIHR peer review process in future. Future research is needed on how best to incorporate feedback from commissioners and policymakers as to how useful the reviews were for decision making as well as potential limitations due to the chosen review methods. There is now a considerable amount of literature available on the use of rapid reviews with details of the methods available and limitations associated with these. There is little guidance on how to choose the most appropriate method for the evidence base identified. It is crucial that commissioners and policy makers have sufficient information to make a judgement on whether or not the chosen review approaches may be considered appropriate and robust. They must also be made aware of what approaches are feasible, bearing in mind the quantity and nature of the evidence as well as the time and resource constraints of the review. Details of the strengths and the limitations of the methods chosen must also be presented to commissioners and policy makers and their potential impact on decision-making. This research goes some way in exploring possible approaches suitable in this context.

## Conclusions

There is no “one size fits all” to the use of rapid review methods. The analyses presented here suggest that the appropriate approach needs to be determined based on the evidence available, time constraints and the needs of policy makers and knowledge users. Authors need to be clear as to what approach was taken and the strengths and limitations of the rapid review methods chosen, how appropriate and robust these choices are and their potential impact on decision-making.

## Abbreviations

HTA: Health technology assessment
NIHR: National Institute for Health Research
PICO: Population, intervention, comparator, outcome
RCT: Randomised controlled trial
